# 

**Published:** 1879-03

**Authors:** 


### Editorial.

### THE NORTHERN OHIO DENTAL ASSOCIATION,

Holds its next annual meeting in Cleveland on Tuesday the 13th of May next, and continues two or three days. This is a well trained society, one that has accomplished much good. It embraces in its membership some of the best men in the dental profession. No one can be present at its meetings without deriving much benefit. The invitation to be present is extended to all. Then let all who possibly can, be present, and good will be the result.

### ILLINOIS DENTAL SOCIETY.

The fifteenth annual meeting of the Illinois State Dental Society will be held in the Representative's Hall, at Springfield, commencing Tuesday, May 13th, 1879, and continue three or four days.

This is one of the most active, efficient and energetic dental societies in existence; the evidences of which are clearly set forth in its annual volume of transactions, from which I have often found it profitable to draw for the pages of the DENTAL REGISTER.

There is always a large attendance at these meetings, and the occasion is always one of interest and profit to those who attend. The invitation extends to all. *Whosoever will, let him come.*

### CALIFORNIA STATE DENTAL ASSOCIATION.

The annual meeting of this society will take place on Tuesday, May 13, 1879, at ten o'clock a. m., and continue four days.

The following subjects are upon the programme for consideration, each of which will be opened by the persons named, respectively:

First. Reflex Influence of Dental Associations and Dental Colleges on the Dental Profession. W. N. Robinson.

Second. Legislation Pertaining to Dentistry. Dr. N. J. Plomteaux.

Third. Conservative Dentistry. R. Cutlar.

Fourth. Periostitis. Cyrus Hamilton.

Fifth. Pulpitis and its Treatment. J. A. W. Lundberg.

Sixth. Dental Legislation. C. E. Davis.

Seventh. Professional Self Reliance. Jno. Rabe.

Eighth. Professional Ethics. J. J. Birge.

Ninth. The Wages of Professional Intercourse. Wm. B. Kingsbury.

Tenth. Mechanical Dentistry Defined. W. C. Harding.

Eleventh. Oral Electricity. J. L. Cogswell.

Twelfth. Professional Culture. G. L. Jenkins.

Thirteenth. Ulitis. C. S. Lane.

These are interesting and important subjects, which ought, and doubtless will, elicit fresh and instructive thoughts and ideas.

The dental profession on the Pacific coast is active and progressive, and in no respect behind the mass of the profession in other portions of the country. It is expected, and desirable, that a large number of the profession be present.

### CONTENTS.

COMMUNICATIONS.

### *THE DENTAL REGISTER,*

A MONTHLY MAGAZINE DEVOTED TO THE INTERESTS OF

### THE DENTAL PROFESSION.

*Terms Reduced to $2 50 per Year. Single Copies 25c.*

### *EDITED BY PROF. J. TAFT, D. D. S.,*

AND PUBLISHED BY

SPENCER & CROCKER, Ohio Dental Depot.

The volume commences in January and closes in December.

The REGISTER being issued monthly is always fresh, therefore Indispensable to the practitioner who desires to keep posted up with the new inventions and improvements which are daily developed. Neither expense nor effort will be spared to give value and variety to its contents. Articles will be illustrated with well executed engravings whenever they will add to the interest or assist to explanation.

Its extensive circulation and frequency of issue make it one of the BEST DENTAL ADVERTISING MEDIUMS in the United States.

All subscrptions must begin with January or July.

Local or special notices, five lines of less, will be inserted for $3.00 each insertion; over five lines, 50 cts. per line.

All advertising bills payable in advance, or at furthest, after the issue of the first number containing the advertisement. All transient advertisements to be paid for in advance.

☞ CONTRIBUTIONS SOLICITED.

Exclusively Editorial Communications should be addressed to Editor.

The latest number of the REGISTER may be ordered with or without other goods at any time, and all letters should be addressed to

**SPENCER & CROCKER, Ohio Dental Depot, Cincinnati, O.**

